# *In vitro *antibacterial activity and acute toxicity studies of aqueous-methanol extract of *Sida rhombifolia *Linn. (Malvaceae)

**DOI:** 10.1186/1472-6882-10-40

**Published:** 2010-07-27

**Authors:** Assam Assam JP, JP Dzoyem, CA Pieme, VB Penlap

**Affiliations:** 1 Department of Biochemistry, Faculty of Science PO Box 812 University of Yaoundé I Cameroon; 2 Department of Biochemistry; Faculty of Science, University of Dschang; PO Box 67 Dschang-Cameroon; 3 Department of Physiological Sciences and Biochemistry, Faculty of Medicine and Biomedical Sciences; PO Box 1364 University of Yaoundé, Yaoundé Cameroon

## Abstract

**Background:**

Many bacteria among the Enterobacteria family are involved in infectious diseases and diarrhoea. Most of these bacteria become resistant to the most commonly used synthetic drugs in Cameroon. Natural substances seem to be an alternative to this problem. Thus the aim of this research was to investigate the *in vitro *antibacterial activity of the methanol and aqueous-methanol extracts of *Sida rhombifolia *Linn (Malvaceae) against seven pathogenic bacteria involved in diarrhoea. Acute toxicity of the most active extract was determined and major bioactive components were screened.

**Methods:**

The agar disc diffusion and the agar dilution method were used for the determination of inhibition diameters and the Minimum Inhibitory Concentration (MICs) respectively. The acute toxicity study was performed according WHO protocol.

**Results:**

The aqueous-methanol extract (1v:4v) was the most active with diameters of inhibition zones ranging from 8.7 - 23.6 mm, however at 200 μg/dic this activity was relatively weak compared to gentamycin. The MICs of the aqueous-methanol extract (1v:4v) varied from 49.40 to 78.30 μg/ml. *Salmonella dysenteriae *was the most sensitive (49.40 μg/ml). For the acute toxicity study, no deaths of rats were recorded. However, significant increase of some biochemical parameters such as aspartate amino-transferase (AST), alanine aminotransferase (ALT), alkaline phosphatase (ALP) and creatinine (CRT) were found. The phytochemical analysis of the aqueous methanol extract indicated the presence of tannins, polyphenols, alkaloids, glycosides, flavonoids and saponins

**Conclusion:**

The results showed that the aqueous-methanol extract of *S. rhombifolia *exhibited moderate antibacterial activity. Some toxic effects were found when rats received more than 8 g/kg bw of extract.

Antibacterial; Enterobacteria; Acute toxicity; Phytochemical analysis

## Background

It has been reported that diarrhoea is the most infant mortality disease in the world, mostly in developing countries. Each year, this disease kills more than 6 million of children in the world with 7.7% and 8.5% respectively in Africa and South East of Asia [1,2]. In Cameroon, diarrhoeic infection is involved in high mortality and morbidity of the children and accounts for thirty thousand deaths among children each year [3]. Another alarming case is the infection by new strain of *Escherichia coli *(O157:H7) which is wide spread in several countries. This strain of *E. coli *has been responsible for different outbreaks of diseases associated with chronic diarrhoea and the dysfunctioning of the kidney which in some cases are lethal [4]. Several researches demonstrated that many strains of Gram-positive and Gram-negative bacteria currently developed outstanding drug resistance marking the search of new, safe, non toxic and effective antibacterial agents become strictly a necessity. Many antibacterial agents are available in the nature for the treatment of systemic infections. Plants therefore constitute good source active agents for this purpose and many plants extracts have been reported to possess various antimicrobial activities [5].

*Sida rhombifolia *is a perennial or sometimes annual plant native to the tropic and subtropic areas. The stems are erect to sprawling and branched growing 50-120 cm in height, with a woody lower section. The dark green, diamond-shaped leaves are arranged alternately along the stem, 4-8 cm long, with petioles that are less than a third of the length of the leaves [6]. *S. rhombifolia *is one of the most important species among the twenty genus of *Sida *used as medicinal plant through out the world. It possesses antiseptic, wound-healing activities and it is also used for the treatment of diarrhoea, cough, ulcer, abscess and furuncle both in Madagascar and in Cameroon [6,7]. In India, infusion of leaves of *S. rhombifolia *has been shown to possess diuretic and aphrodisiac effects. It is also used there for the treatment of dysenteriae, tuberculosis, skin, urogenital diseases and also as food [8]. The root and stems of the plant are useful for the treatment of fever, heart disease, piles and some inflammations [9]. A stem of S. *rhombifolia *is also employed as demulcent and emollient [10]. Various studies reported that the methanol extract of *S. rhombifolia *showed anti-tumor and anti-HIV activities [11]. The inhibitory activity of the ethanol extract of *S. rhombifolia *on NF-Kb cell line as anti-inflammatory model was demonstrated [12]. Numerous studies investigated on various organic extracts of *S. rhombifolia *have shown the antinociceptive, anti-inflammatory, cytotoxicity, antibacterial and larvicidal activities [13,9,14]. Although *S. rhombifolia *is widely used for the above mentioned biological activities, no toxicological study of the plant has been reported previously as well as its *in vitro *anti-diarrhoea activity in Cameroon. As a contribution to the search of non toxic, novel antibacterial principle from medicinal plants of Cameroon, results of *in vitro *antibacterial investigation is being reported herein.

## Methods

### Chemicals

Gentamicin usually used was acquired from a local pharmacy. Methanol, chloroform, nutrient agar and nutrient broth were purchased from Merck Company and other chemicals used were from Sigma Company.

### Plant material

*Sida rhombifolia *was collected in June 2004 at *Bidjom *locality in the south region of Cameroon. Botanical identification was made by Mr P. Mezili of the Cameroon National Herbarium and the voucher specimen was deposited under the number SA/2004HN.

#### Preparation of extract

The collected plant was dried at room temperature (30 ± 3°C), pulverized and sieved. For the extraction, the powders of 25 g each were separately macerated in four solvents for 72 h. These solvents included 500 ml of methanol and the same volume of three mixtures of water and methanol (1v:4v), (1v:1v), (3v:2v). Each solution was filtered using Whatman filter paper N°1 and concentrated in an air circulating oven at 54°C until total dryness. Four crude extracts denoted A, B, C, D were obtained corresponding respectively to MeOH extract; water/MeOH (1v:4v); (1v:1v) and (3v:2v) extracts. The experiment was repeated twice with the same powder for each solvent and the crude extracts obtained were stored at 5°C. For the antibacterial assay, a stock solution (50 mg/ml) was prepared for each extract by dissolving the extract in water.

### Antibacterial assay

#### Microorganisms

Seven species of multi-resistant enterobacteria strains isolated from patients at "*Centre Pasteur of Cameroon" *were used for the evaluation of antibacterial activity. All these micro-organisms were Gram-negative and included *Escherichia coli, Proteus vulgaris, Morganella morganii, Salmonella typhi, Shigella dysenteriae Salmonella enteritidis *and *Klebsiella pneumoniae*.

#### Inocula preparation

An inoculum for each micro-organism was prepared from broth cultures containing approximately 5.10^5 ^to 9.10^6 ^colony forming units per mililiter (CFU/ml). Each diluted (1:50) inoculum was applied as a lawn with a micropipette calibrated to deliver 50 μl containing around 9.10^6 ^CFU. The discs impregnated with each extract were evaporated for 24 hours. Each disc containing 500 μg of extract was then deposited on the culture of micro-organisms. The plates were incubated for 20 h at 37°C.

#### Determination of the diameters of inhibition zone

The four crude extracts (A, B, C, D) were tested *in vitro *for antibacterial activity by the standard disc diffusion method against the micro-organisms at 500 μg extract per disc [15]. Gentamycin used as standard antibiotic (positive control) was tested at 133 μg/disc. The diameters of inhibition zones produced by these extracts were then compared to standard antibiotic (Gentamycin). Extract B water/MeOH (1v:4v) which demonstrated the highest activity was tested at 50, 100, 150 and 200 μg/disc using the same method.

#### MIC determination

For MICs determination, only the most sensitive microorganisms (*P. vulgaris, K. pneumoniae, S. dysenteriae*) were tested at 100 μg/disc with extract B. Serial dilutions were set up with 10, 20, 40, 60, 80 and 100 μg/ml of water/MeOH (1v:4v) extract in the nutrient broth medium. 200 μl of the suspension of each pathogenic bacterium (9 × 10^7 ^cell/ml) were added and incubated at 37°C for 24 h. The lowest concentration which did not show any macroscopic growth of tested micro-organism was identified as the MIC.

### Toxicity study

#### Experimental animals

Albino *Wistar *rats (102-134 g) of about two and half months were obtained from the animal laboratory of the Biochemistry Department of the Yaounde I University, Cameroon. All the rats were kept under environmental conditions (27 ± 2°C) and they had free access to water and food. These rats were deprived of food but not water (16-18 h) prior to the administration of the extract. The principles of laboratory animal care were followed while the Department's ethical committee approved the use of the animals and the study design.

#### Acute toxicity

The bioassay was conducted according to the World Health Organisation guideline for the evaluation of safety and efficacy of herbal medicines [16]. For the study, albino *Wistar *rats were divided into five groups of six animals each. The aqueous-methanol extract (1v:4v) of *S. rhombifolia *was suspended in the distilled water. This extract was employed because of its moderate or high antibacterial activity against all the bacteria considered. This solution was administrated *per os *to rats groups (2-5) in a single oral dose of 4, 8, 12 and 16 g/kg body weight (b w) respectively by intra gastric gavages using a feeding needle. The control group received an equal volume of distilled water as vehicle. Observations of toxic symptoms were made and recorded systematically 1, 2, 4, 6 and 24 h after administration of the extract. The number of rats that survived were noted after 24 h and then maintained for the further 8 days with daily observations. This visual observation included skin changes, mobility, and aggressiveness, sensitivity to sound and pain, as well as respiratory movements. The toxic effects of the extract were assessed on the basis of mortality, which was expressed as LD_50 _[17]. During the experiment, the animals were weighed, food and water intake were monitored. At the end of the experiment, all surviving animals fasted overnight and sacrificed by decapitation. The organs such as liver, lungs, heart and kidneys were excised and weighed. The pathological observations of these tissues were performed on gross. The blood samples were also collected freshly in the dry non-heparinised centrifuge tubes. This blood was allowed to coagulate before being centrifuged to separate the serum. This serum was assayed for biochemical parameters. The liver was excised, rinsed in ice-normal solution followed by cold 0.1 M Tris-HCl (pH 7.5), blotted, dried and weighed. The 20% (w/v) liver homogenates was prepared in the 0.1 M Tris-HCl buffer and the supernatant was used for biochemical analysis. Lung, kidney and heart were removed, washed in 0.9% NaCl weighed and examined as previously mentioned.

#### Determination of biochemical parameters

Blood collected into non heparinised tubes was centrifuged at 3000 rpm for 10 min to separate the serum. This serum was used to evaluate the liver enzyme function through some biochemical parameters such as aspartate aminotransferase (AST), alanine aminotransferase [18] alkaline phosphatase [19,20]; creatinine [21] and protein [22]. For the 20% liver homogenates the same parameters were assayed only the creatinine was replaced by the glutathione [23].

### Phytochemical screening

Qualitative phytochemical tests were carried out to identify some components of aqueous-methanol (1v:4v) extract of *S. rhombifolia *[24-26]. The plant was screened for alkaloids, saponins, tannins, flavonoids, polyphenols, anthraquinones.

*Test for alkaloids*: 0.5 g of the sample was stirred with 5 ml of 1% aqueous HCl on a steam bath and then filtered. 1 ml of the filtrate was treated with a few drops of Mayer's reagent and a second 1 ml portion was treated similarly with Dragendroff reagent. Turbidity or precipitation with either of these reagents was taken as evidence for the presence of alkaloids in the extract [24].

*Test for saponins: *The ability of saponins to produce frothing in aqueous solution and to haemolyse red blood cells was used for the screening test. 0.5 g of plant extract was shaken with water in a test tube. Frothing which persisted on warming was taken as evidence for the presence of saponins [24].

*Test for tannins*: 0.5 g of dried extract was stirred with 5.0 ml of distilled water. This was filtered and ferric chloric reagent was added to the filtrate. A blue-black precipitate was taken as evidence for the presence of tannins [24].

*Test for polyphenols*: 2 ml of plant extract was heated for 30 min in a water bath. 1 ml of 1.00% FeCl_2 _was added to the mixture then followed by the addition of 1 ml of 1.00% potassium ferrocyanide. The mixture was filtered and the formation of a green-blue colour indicated the presence of polyphenol [25].

*Test for anthraquinones*: 0.5 g of plant extract was shaken with 5 ml of benzene, filtered and 2 ml of 10% ammonia solution was added to the filtrate. The mixture was shaken and the presence of a pink or violet colour in the ammoniacal (lower) phase indicated the presence of free hydroxy anthraquinones [26].

#### Statistical analysis

The values were expressed as mean ± standard deviation (SD). Each value was a mean of five or six tests. The one-way analysis of variance (ANOVA) was used to determine the significant differences between parameters and the student-Newman Keurls test served to compare these differences at *p *< 0.05 using statistical package used was SPSS 10.1.

## Results

### Antibacterial activity

The results of the *in vitro *antibacterial activity of methanol and aqueous methanol extracts determined by diameters of inhibition zones are presented in Additional file 1. These results indicated that the diameters of inhibition zones varied from 8.7 - 23.6 mm and 19.4 - 26.5 for the extracts and gentamycin respectively. Among the four extracts, aqueous-methanol (1v:4v) extract was the most active with 23.6 mm of inhibition diameter against *S. dysenteriae*. Gentamycin used as a standard antibiotic at the concentration of 133 μg/disc exhibited higher diameters of inhibition than other extracts. The solvent dilution of the extract showed any antibacterial activity. These two extracts (MeOH and water/MeOH (1v:4v)) revealed interesting antibacterial activities on six of the seven enterobacteria tested. The aqueous-methanol extract (1v:4v) was most active than other extracts. All the extracts showed moderate or higher activities against *S. typhi *and *S. dysenteriae*. The aqueous-MeOH extracts (1v:1v); (3v:2v) demonstrated antibacterial properties only on 3 and 4 bacteria used in the experiment. Both of them exhibited high and moderate antibacterial activities against *S. dysenteriae *respectively with extracts C and D.

Additional file 2 shows the results of aqueous-MeOH extract (1v:4v) of *S. rhombifolia *on seven enterobacteria strains. These results revealed that the diameters of inhibition zones increased with the concentration of extract. In the range of 100 - 200 μg/disc, the extract exhibited antibacterial activity against all the bacteria strains used except *S. enteritidis*. Among the seven isolates, four bacteria (*P. vulgaris, S. typhi, S. dysenteriae and K. pneumoniae) *were sensitive to the extracts at all the concentrations. However, *S. typhi *was the most sensitive at the lowest concentration (50 μg/disc) of the extract. The highest diameter of inhibition zone (24.1 mm) was found with *S. dysenteriae *at 200 g μg/disc. *S. enteritidis *was the most resistant bacteria isolate with diameters of inhibition zones ranging between 8.01 - 8.5 mm. The results of the antibacterial activity of the most active extract determined by MICs are represented in Additional file 3. The MICs of aqueous-MeOH ranged between 49 and 78 μg/ml. These results revealed that *S. dysenteriae and K. pneumoniae *were the most sensitive with values of 50.00 μg/ml and 49.40 μg/ml respectively (Additional file3). The MICs of the extract of *S. rhombifolia *were 7.8; 4.5 and 3.1 fold less active than standard antibiotic (gentamycin).

### Acute toxicity

For the acute toxicity studies, no death of rats was neither recorded in the control nor in the treated groups. The animals exhibited slight changes in general behaviour (slow response to external stimuli, stretching and sluggishness) but did not expressed changes in their physiopatological activities. Although there was an increase in the weights of rats and their organs in the treated groups, this addition remained statistically not significant compare with control (Additional files 4 and 5). The pathological examinations of the tissues on a gross basis indicated no detectable abnormalities at the end of the experiment. Additional file 6 shows the results of the blood and hepatic parameters. These results indicated that alkaline phosphatase (ALP), alanine aminotransferase (ALT), aspartate aminotransferase (AST), creatinine (CRT) of the treated rats increased significantly (*p *< 0.05) at the higher concentration of the extract compare to the control. Unlike the protein concentration and AST activity, the ALT and ALP activities were higher in the liver than in the blood.

### Phytochemical screening

The phytochemical screening of the extract of *S. rhombifolia *indicated the presence of three main classes of compounds: polyphenols, alkaloids and steroids. The polyphenols group constitutes the principal component of *S. rhombifolia *extract with three bioactives subgroups: tannins, flavonoids and saponins. Anthraquinone was absence in the extract of *S. rhombifolia*. Alkaloids and steroids whose antimicrobial activities have been demonstrated were also found in this extract.

## Discussion

This work permitted the evaluation of some biological properties of *S. rhombifolia*; among which the antimicrobial activity, directed on some pathogens frequently encountered in diarrheoic infections as well as the toxicological study of this extract. The diameters of inhibition zones of MeOH, water/MeOH (1v:4v), water/MeOH (1v:1v) and water/MeOH (3v:2v) extracts ranged between 9.5 - 18.5; 8.7 - 23.6; 10.2 - 19.2 and 9.6 - 15.6 respectively. The inhibition diameters can be classified into four categories [27]: No activity (diameter of inhibition < 7 mm); weak activity (diameter of inhibition between 7 mm and 10 mm), moderate activity (diameter of inhibition between 11 mm and 16 mm) and good or higher activity (diameter of inhibition between > 16 mm). The results of antimicrobial activity of *S. rhombifolia *demonstrated that the aqueous-methanol extract (1v:4v) exhibited moderate or higher inhibition zones than other extracts depending of the microorganisms. The presence of water in small quantity facilitates the solubility of hydro-soluble bio-active components and enhances their extraction. It was also observed in overall results of Additional file 1 that, the antibacterial activity decrease when the proportion of water increase in the mixture solvent. In fact water and palm wine are the solvents commonly used in the folk medicine in Cameroon. Methanol is widely used in extraction for scientific purpose. The methanol extract of *S. rhombifolia *demonstrated weak activity compared to the aqueous-MeOH (1v:4v) extract. Probably the small proportion of water in the mixture solvent (1v:4v) may increased the quality or the quantity of the phytochemical bio-active components in this extract [28]. All the micro-organisms tested here were harmful and involved in diarrhoea and other infectious diseases. Some authors have reported the antibacterial activities of the petroleum ether, chloroform, ethyl-acetate extracts of *S. rhombifolia *on Gram-positive (*B. subtilis, B. megaterium, S. aureus and S. lutea*) and Gram-negative bacteria (*E. coli, Klebsiella. Sp., P. aeruginosa, S. dysenteriae, S. sonnei and S. boydii *[29]. The broad antimicrobial activity of the extract (1v:4v) in our study can be attributed to the presence of various bio-actives components such as tannins, polyphenols, alkaloids, glycosides, flavonoids, steroids and saponins found in this extract. Two glycosides (phenyl-Ethyl-D-glucopyranoside and Phytoécdysteroide) exhibited different biological activities were isolated from the extract of the seeds of *S. rhombifolia *respectively [28,30]. The variation observed of the diameters of inhibition zone of the bacteria tested can be attributed either to the difference in the composition of the bio-active molecules present in the extract or to their mechanism of action on Gram-positive and Gram-negative bacteria. The mechanism of action of the glycosides, polyphenols, tannins and alkaloids on Gram-positive and Gram-negative bacteria was demonstrated [28]. The MICs of the most sensitive isolate to the aqueous-methanol extract (1v:4v) varied from 49.40 to 78.30 μg/ml. The antibacterial property of *S. dysenteriae and K. pneumoniae *is well documented [31,32]. Several studies reported that *S. rhombifolia *is traditionally used to treat gonorrhoea (India) and diarrhoea (Cameroon) [6,33]. The anti-inflammatory and hepato-protective activities of the methanol extract of the aerial parts and aqueous extract of the roots and aerial parts of *S. rhombifolia *have been demonstrated [33]. Both cytotoxicity and antibacterial activities of the ethyl acetate of extract of *S. rhombifolia *have been established while its methanol extract exhibited *in vitro *cytotoxic activities against 6 human cell lines [14,34]. Other studies revealed the antimicrobial, anti-HIV and anti-tumor activities of extract of *S. rhombifolia *in Bangladesh [10,11,30]. The acute toxicity test has been investigated to establish the adverse effects of the administration of the aqueous-methanol extract of *S. rhombifolia *on some behavioural and biochemical parameters. The results indicated that, up to 16 g/kg bw, no abnormal symptoms and no death of the rats was observed. According to the OCED protocol [35]*S. rhombifolia *extract can be classified as non toxic since the limited dose of an acute toxicity is generally considered to be 5.0 g/kg bw [17]. If no mortality is observed at this level, a higher dosage is generally not necessary [36]. However, the body weight of the rats increased during the experiment. Compared to the control, the weight gained by the treated rats was higher but statistically not significant. This result showed that the extract slightly stimulates the appetite of the rats and probably not irritates directly the gastrointestinal tract. However, the increase of the weight of the heart, kidneys, and liver after the administration of high dose of extract can be an indicator of some toxic effects. In this way, the significant changes noted in some of the blood chemistry parameters such as ALT, AST, ALP and CRT were undoubted. It should be noted that these modifications were observed at high dose (more than 8 g/kg bw). Even though the changes noted were not significant, they were statistically different compare to control. The variation of biochemical parameters indicated the malfunctioning of one or many organs. The increase of the activity of ALP after the administration of the extract may indicate the obstruction of the bile duct. However, this variation can not be attributed only to a dysfunction of the bile duct since many sources of ALP are known (liver cells; osteoblast, intestinal cells and placenta tissue). Although the variation of ALT and AST activities are associated with the hepato-cellular damage, only ALT is specific for the evaluation of liver damage. AST is highly concentrated in cardiac muscle, liver, skeletal muscle and kidneys. The significant increase of ALT and AST activities after treatment of the rats with 12 and 16 g/kg bw of extract implies an injury of the liver as well as the heart or other sources of these enzymes. The creatinine level of treated rats increased but remained less than one-fold greater compare with the control. This last result confirms that kidneys are slightly affected at the dose of 16 g/kg body weight of the extract. The variation of other parameters such as protein and glutathione were not statistically significant. It has been recognized that eventual therapeutic bioactive products from plants may also contain substances which act as poisons in human [29]. Several researches demonstrated that phenolics and polyphenols compounds have antimicrobial activities [28]. Other workers have shown that the sites and the number of hydroxyl groups on the phenol are thought to be related to their relative toxicity to microorganisms, with the evidence that an increase of hydroxylation will result to an increase toxicity [28,37,38]. The presence of three subclasses of polyphenols (tannins, flavonoids and saponins) in the extract may have significant inhibitory effect on all isolates tested. The antibacterial effects of each of these subclasses of polyphenols on Gram-positive and Gram-negative have been demonstrated as well as the microbicidal effects of alkaloids [39,40]. The presence of multiple phytochemical components confers to *S. rhombifolia *extracts its *in vitro *antibacterial activity.

## Conclusion

The aqueous-methanol (1v:4v) extract of *S. rhombifolia *demonstrated effective *in vitro *antibacterial activity. However some toxic effects have been discovered after administration of high dose of this extract. Further research needs to be carried out to identify the active molecules and evaluate the *in vivo *antibacterial activity as well as sub-acute or chronic toxicities.

## Competing interests

The authors declare that they have no competing interests.

## Authors' contributions

AAJP carried out the study; PCA provided assistance in the investigation of the antimicrobial and acute toxicity, provided the plant, prepared the manuscript; DJP provided chemical and assistance for toxicological studies, participated in the preparation of the manuscript; PBV supervised the work, evaluated the data, corrected the manuscript for publication and coordinated the study. All the authors read and approved the final manuscript.

## Pre-publication history

The pre-publication history for this paper can be accessed here:

http://www.biomedcentral.com/1472-6882/10/40/prepub

## Supplementary Material

Additional file 1 **Table s1: Diameters of inhibition zone of different extracts of S. *rhombifolia *Linn**. This table contents the diameters of inhibition zone of different extracts: MeOH,; water/MeOH (1v:4v); water/MeOH (1v:1v); water/MeOH (3v:2v); Gent: Gentamycin. All the extracts are tested at 500 μg/dic and 133 μg/disc for gentamicin. Click here for file

Additional file 2 **Table s2: Diameters of inhibition zone of different concentrations of aqueous methanol (1v:4v) of S. rhombifolia on bacteria**. The table shows the diameters of inhibition zone of aqueous-methanol extract (1v:4v) of *S. rhombifolia *at the concentrations (50 μg/dic, 100 μg/dic, 150 μg/dic, 200 μg/dic) Click here for file

Additional file 3 **Table s3: Minimum inhibitory concentration of aqueous methanol extract (1v:4v) of *S. rhombifolia *on the parameter of inhibition of bacteria**. This table shows the MICs of extract of S. rhombifolia against most sensitivebacteria (*P. vulgaris, K. pneumonia, S. dysenteriae*). Click here for file

Additional file 4 **Table s4: Body weights of rats after 8 days of acute toxicity of aqueous methanol extract of *S. rhombifolia *Linn**. Variation of body weight of five groups of rats during acute toxicity assay. Click here for file

Additional file 5 **Table s5: Weights organs of rats in the acute toxicity of aqueous methanol extract of *S. rhombifolia *Linn**. Variation of weight of organs (heart, lung, kidney and liver) 8 days after administration of unique dose of extract (acute toxicity) Click here for file

Additional file 6 **Table s6: Blood and liver homogenates biochemical index of rats after acute toxicity of aqueous-methanol extract of S. *rhombifolia *Linn**. This table shows the results of biochemical parameters (ALAT, AST, ALP, Creatinine, protein) for serum and liver homogenates of five groups of rats after 8 days of administration of extract. Click here for file
