# Supplementary material for: In vitro antibacterial activity and acute toxicity studies of aqueous-methanol extract of Sida rhombifolia Linn. (Malvaceae)
Source: BMC Complement Altern Med. 2010 Jul 27;10:40. doi: 10.1186/1472-6882-10-40 (PMC2922083; doi:10.1186/1472-6882-10-40)
Supplement: Additional file 1 — Table s1: Diameters of inhibition zone of different extracts of S. rhombifolia Linn. This table contents the diameters of inhibition zone of different extracts: MeOH,; water/MeOH (1v:4v); water/MeOH (1v:1v); water/MeOH (3v:2v); Gent: Gentamycin. All the extracts are tested at 500 μg/dic and 133 μg/disc for gentamicin. [file 1472-6882-10-40-S1.DOC]

**Additional file 1: DOA**

**Table1: Diameters of inhibition zone of different extracts of S*. rhombifolia* Linn.**

**Description**: This table contents the diameters of inhibition zone of different extracts: MeOH, ; water/ MeOH (1v:4v); water /MeOH (1v:1v); water/MeOH (3v:2v); Gent: Gentamycin. All the extracts are tested at 500 µg/dic and 133 µg/disc for gentamicin.

**Table1: Diameters of inhibition zone of different extracts of S*. rhombifolia* Linn**.

| Bacteria species | **Diameters of inhibition (mm)** | | | | |
| --- | --- | --- | --- | --- | --- |
|  | **A** | **B** | **C** | **D** | **Gent** |
| ***E. coli*** | 11.5 ± 0.1 | 11.5 ± 0.8 | 10.2 ±1.0 | 9.6 ±0.2 | 23.0 ± 0.6 |
| ***P. vulgaris*** | 9. 5 ± 0.2 | 15.1 ± 0.3 | 12.2 ± 0.2 | N | 26.2 ± 0.7 |
| ***M. morganii*** | 11.2 ± 0.4 | 11.4 ± 0.2 | N | N | 19.4 ± 0.5 |
| ***S. typhi*** | 12.5 ± 0.8 | 15.8 ± 0.2 | 13.3 ± 0.7 | 10.3 ± 0.5 | 26.5 ± 0.5 |
| ***S. enteritidis*** | 14.1 ± 0.3 | 8.7 ± 0.9 | N | 15.6 ± 0.6 | 22.2 ± 0.4 |
| ***S. dysenteriae*** | 11.5 ± 0.4 | 23.6 ±0.8 | 21.2 ± 0.5 | 14.3 ± 0.6 | 22.5± 0.7 |
| ***K****. pneumoniae* | 18.5 ± 0.4 | 19.3 ± 0.7 | 19.2 ±1.3 | N | 26.5 ± 0.6 |

A: MeOH; B: water/ MeOH (1v:4v); C: water /MeOH (1v:1v); D: water/MeOH (3v:2v); Gent: Gentamycin; N: diameter < 8 mm; Values are expressed as mean ± SD, (n = 3).
