# Supplementary material for: In vitro antibacterial activity and acute toxicity studies of aqueous-methanol extract of Sida rhombifolia Linn. (Malvaceae)
Source: BMC Complement Altern Med. 2010 Jul 27;10:40. doi: 10.1186/1472-6882-10-40 (PMC2922083; doi:10.1186/1472-6882-10-40)
Supplement: Additional file 2 — Table s2: Diameters of inhibition zone of different concentrations of aqueous methanol (1v:4v) of S. rhombifolia on bacteria. The table shows the diameters of inhibition zone of aqueous-methanol extract (1v:4v) of S. rhombifolia at the concentrations (50 μg/dic, 100 μg/dic, 150 μg/dic, 200 μg/dic) [file 1472-6882-10-40-S2.DOC]

**Additional file 2: DOB**

**Table 2: Diameters of inhibition zone of different concentrations of aqueous methanol (1v:4v) of S. rhombifolia on bacteria.**

**Description:** The table shows the diameters of inhibition zone of aqueous-methanol extract (1v:4v) of *S. rhombifolia* at the concentrations (50 µg/dic, 100 µg/dic, 150 µg/dic, 200 µg/dic)

**Table 2: Diameters of inhibition zone of different concentrations of aqueous methanol (1v:4v) of S. rhombifolia on bacteria.**

| Bacteria species | **Diameters of inhibition (mm)** | | | | |
| --- | --- | --- | --- | --- | --- |
|  | **50 (µg/disc)** | **100 (µg/disc)** | **150 (µg/disc)** | **200 (µg/disc)** | **Gent 133 (µg/disc)** |
| ***E. coli*** | 9.33 ± 0.75 | 10.33 ± 0.60 | 11.50 ± 0.60 | 11.80 ± 1.04 | 23.00 ± 0.06 |
| ***P. vulgaris*** | 11.20 ±1.01 | 14.30 ± 0.57 | 15.66 ± 0.33 | 16.20 ± 0.57 | 26.00 ± 0.00 |
| ***M. morganii*** | ND | 8.00 ± 0.50 | 10.16 ± 0.60 | 11.50 ± 0.28 | 19.00 ± 0.00 |
| ***S. typhi*** | 11.45 ± 0.07 | 14.00 ± 0.57 | 14.30 ± 0.58 | 16.03 ± 0.10 | 26.50 ± 0.00 |
| ***S. enteritidis*** | ND | 8.01 ±0.28 | 8.30 ± 0.50 | 8.50 ± 0.28 | 22.00 ±0.00 |
| ***S. dysenteriae*** | 11.02 ± 0.10 | 15.10 ± 0.57 | 18.00 ±0.57 | 24.10 ± 0.50 | 22.50 ± 0.00 |
| ***K. pneumoniae*** | 11.00 ± 0.76 | 14.10 ± 0.30 | 15.20 ± 0.06 | 19.50 ± 0.50 | 26.00 ± 0.00 |

Gent: Gentamycin; ND: diameter <8 mm; Values are expressed as mean ± SD (n = 3).
