# Supplementary material for: In vitro antibacterial activity and acute toxicity studies of aqueous-methanol extract of Sida rhombifolia Linn. (Malvaceae)
Source: BMC Complement Altern Med. 2010 Jul 27;10:40. doi: 10.1186/1472-6882-10-40 (PMC2922083; doi:10.1186/1472-6882-10-40)
Supplement: Additional file 3 — Table s3: Minimum inhibitory concentration of aqueous methanol extract (1v:4v) of S. rhombifolia on the parameter of inhibition of bacteria. This table shows the MICs of extract of S. rhombifolia against most sensitivebacteria (P. vulgaris, K. pneumonia, S. dysenteriae). [file 1472-6882-10-40-S3.DOC]

**Additional file 3: DOC**

**Table 3: Minimum inhibitory concentration of aqueous methanol extract (1v:4v) of *S. rhombifolia* on the parameter of inhibition of bacteria**

**Description:** This table shows the MICs of extract of S. rhombifolia against most sensitivebacteria (*P. vulgaris, K. pneumonia, S . dysenteriae*).

**Table 3: Minimum inhibitory concentration of aqueous methanol extract (1v:4v) of *S. rhombifolia* on the parameter of inhibition of bacteria**

| Bacteria species | **Parameters of inhibition** | |
| --- | --- | --- |
|  | Extract of *S. rhombifolia* | Gent. |
|  | MIC (µg/ml) | MIC (µg/ml) |
| *P .vulgaris* | 78.30 ± 0.10 | 10.16 ± 0.16 |
| *K. pneumoniae* | 50.00 ±0.00 | 16.66 ± 3.33 |
| *S .dysenteriae* | 49.40 ±0.30 | 10.16±0.16 |

Gent: Gentamycin; Values are expressed as mean ± SD, (n = 3).
