# Supplementary material for: In vitro antibacterial activity and acute toxicity studies of aqueous-methanol extract of Sida rhombifolia Linn. (Malvaceae)
Source: BMC Complement Altern Med. 2010 Jul 27;10:40. doi: 10.1186/1472-6882-10-40 (PMC2922083; doi:10.1186/1472-6882-10-40)
Supplement: Additional file 4 — Table s4: Body weights of rats after 8 days of acute toxicity of aqueous methanol extract of S. rhombifolia Linn. Variation of body weight of five groups of rats during acute toxicity assay. [file 1472-6882-10-40-S4.DOC]

**Additional file 4: DOD**

**Table 4: Body weights of rats after 8 days of acute toxicity of aqueous methanol extract of *S. rhombifolia* Linn*.***

**Description:** Variation of body weight of five groups of rats during acute toxicity assay.

***Table 4: Body weights of rats after 8 days of acute toxicity of aqueous methanol extract of S. rhombifolia Linn***.

| **Treatment (g/kg)** | **Body weight (g)** | | |
| --- | --- | --- | --- |
|  | **Day 0** | **Day 8** | **Weight gained** |
| Control | 117.12 ± 22.16 | 141.49 ± 25.69 | 24.35 ± 9.51 |
| 4 | 123.37± 47.25 | 163.13 ± 47.75 | 35.79 ± 8.71 |
| 8 | 139.01± 53.19 | 168.67 ± 60.11 | 29.69 ± 11.17 |
| 12 | 136.76 ± 42.00 | 164.22 ± 42.77 | 27.64 ± 14.52 |
| 16 | 132.48 ± 28.09 | 158.26 ± 29.25 | 25.75 ± 12.68 |

Values are expressed as mean ± SD; (n = 5); No significant different (*P*<0.05).
