# Supplementary material for: In vitro antibacterial activity and acute toxicity studies of aqueous-methanol extract of Sida rhombifolia Linn. (Malvaceae)
Source: BMC Complement Altern Med. 2010 Jul 27;10:40. doi: 10.1186/1472-6882-10-40 (PMC2922083; doi:10.1186/1472-6882-10-40)
Supplement: Additional file 5 — Table s5: Weights organs of rats in the acute toxicity of aqueous methanol extract of S. rhombifolia Linn. Variation of weight of organs (heart, lung, kidney and liver) 8 days after administration of unique dose of extract (acute toxicity) [file 1472-6882-10-40-S5.DOCX]

**Additional file 5: DOE**

**Table 5: Weights organs of rats in the acute toxicity of aqueous methanol extract of *S. rhombifolia* Linn.**

**Description:** Variation of weight of organs (heart, lung, kidney and liver) 8 days after administration of unique dose of extract (acute toxicity)

**Table 5: Weights organs of rats in the acute toxicity of aqueous methanol extract of *S. rhombifolia* Linn.**

| **Treatment (g/kg)** | **Weight of organs (g)** | | | |
| --- | --- | --- | --- | --- |
|  | **Heart** | **Lungs** | **Kidneys** | **Liver** |
| Control | 0.56 ± 0.04 | 1.47 ± 0.12 | 1.05 ± 0.14 | 4.32 ± 0.42 |
| 4 | 0.57± 0.10 | 1.16 ± 0.34 | 1.21± 0.40 | 5.60 ± 1.74 |
| 8 | 0.68 ± 0.18 | 1.40 ± 0.17 | 1.44 ± 0.37 | 6.08 ± 1.98 |
| 12 | 0.61± 0.07 | 1.26 ± 0.21 | 1.24 ± 0.22 | 5.85 ± 1.59 |
| 16 | 0.61 ± 0.07 | 1.46 ± 0.36 | 1.13 ± 0.26 | 5.66 ± 1.31 |

Values are expressed as mean ± SD; (n = 5); No significant different (*P*<0.05).
