# Supplementary material for: In vitro antibacterial activity and acute toxicity studies of aqueous-methanol extract of Sida rhombifolia Linn. (Malvaceae)
Source: BMC Complement Altern Med. 2010 Jul 27;10:40. doi: 10.1186/1472-6882-10-40 (PMC2922083; doi:10.1186/1472-6882-10-40)
Supplement: Additional file 6 — Table s6: Blood and liver homogenates biochemical index of rats after acute toxicity of aqueous-methanol extract of S. rhombifolia Linn. This table shows the results of biochemical parameters (ALAT, AST, ALP, Creatinine, protein) for serum and liver homogenates of five groups of rats after 8 days of administration of extract. [file 1472-6882-10-40-S6.DOC]

**Additional file 6: DOF**

**Table 6: Blood and liver homogenates biochemical index of rats after acute toxicity of aqueous-methanol extract of S*. rhombifolia* Linn.**

**Description:** This table shows the results of biochemical parameters (ALAT, AST, ALP, Creatinine, protein) for serum and liver homogenates of five groups of rats after 8 days of administration of extract.

**Table 6: Blood and liver homogenates biochemical index of rats after acute toxicity of aqueous-methanol extract of S*. rhombifolia* Linn.**

| **Biochemical parameters** | | | | | | | | | | |
| --- | --- | --- | --- | --- | --- | --- | --- | --- | --- | --- |
|  | **Serum** | | | | | **Liver homogenate** | | | | |
| **Dose** (**g/kg)** | **ALT** (UI/L**)** | **AST** (UI/L**)** | **ALP** (UI/L**)** | **CRT**(mg/ml**)** | **PR.** (mg/ml) | **ALT** (UI/L**)** | **AST** (UI/L**)** | **ALP** (UI/L**)** | **GSH** (mM/mg) | **PR** (mg/ml**)** |
| Control | 40.09±1.71 | 42.10±1.92^*^ | 93.65±3.00 | 2.68± 0.41^*^ | 0.52±0.14 | 77.64±1.15 | 29.41±1.61 | 98.92±2.21 | 3.39±0.38 | 0.24±0.12 |
| 4 | 38.86±0.42 | 32.75±1.72 | 129.22±4.70^*^ | 2.23± 0.61 | 0.32±0.01 | 82.02±1.16^*^ | 49.25±1.25^*^ | 191.01±4.32^*^ | 4.23±0.68 | 0.31± 0.10 |
| 8 | 43.37±1.38^*^ | 37.32±0.65^*^ | 135.70±1.67^*^ | 2.11± 0.65 | 0.38±0.08 | 89.70±2.64^*^ | 84.64±0.68^*^ | 245.06±1.75^*^ | 5.98±2.52 | 0.20±0.21 |
| 12 | 47.13±2.23^*^ | 45.16±1.25^*^ | 220.54±2.56^*^ | 2.50± 0.89^*^ | 0.40±0.07 | 98.91± 0.69^*^ | 74.29±1.12^*^ | 279.56±5.62^*^ | 6.28±0.77 | 0.18±0.01 |
| 16 | 52.26±1.42^*^ | 47.33±0.66^*^ | 233.36±3.65^*^ | 2.90± 0.74^*^ | 0.35±0.05 | 93.46±1.00^*^ | 120.24±3.12^*^ | 284.06±1.58^*^ | 7.45±1.25 | 0.15±0.02 |

GSH: Glutathione; PR: Protein; CRT: Creatinine; Values are expressed as mean ±SD; n= 6; *Significant from control (*P*<0.05).
